# Recent Highlights in Sustainable Bio-Based Edible Films and Coatings for Fruit and Vegetable Applications

**DOI:** 10.3390/foods13020318

**Published:** 2024-01-19

**Authors:** Valter F. R. Martins, Manuela E. Pintado, Rui M. S. C. Morais, Alcina M. M. B. Morais

**Affiliations:** CBQF—Centro de Biotecnologia e Química Fina—Laboratório Associado, Escola Superior de Biotecnologia, Universidade Católica Portuguesa, Rua Diogo Botelho, 1327, 4169-005 Porto, Portugal; s-vfrmartins@ucp.pt (V.F.R.M.); mpintado@ucp.pt (M.E.P.); rcmorais@ucp.pt (R.M.S.C.M.)

**Keywords:** biological resources, edible coatings, edible films, packaging, bioactive compounds, antimicrobial activity, antioxidant activity, fruits, vegetables, biodegradability

## Abstract

The present review paper focuses on recent developments in edible films and coatings made of base compounds from biological sources, namely plants, animals, algae, and microorganisms. These sources include by-products, residues, and wastes from agro-food industries and sea products that contribute to sustainability concerns. Chitosan, derived from animal biological sources, such as crustacean exoskeletons, has been the most studied base compound over the past three years. Polysaccharides typically constitute no more than 3–5% of the film/coating base solution, with some exceptions, like Arabic gum. Proteins and lipids may be present in higher concentrations, such as zein and beeswax. This review also discusses the enrichment of these bio-based films and coatings with various functional and/or bioactive compounds to confer or enhance their functionalities, such as antimicrobial, antioxidant, and anti-enzymatic properties, as well as physical properties. Whenever possible, a comparative analysis among different formulations was performed. The results of the applications of these edible films and coatings to fruit and vegetable products are also described, including shelf life extension, inhibition of microbial growth, and prevention of oxidation. This review also explores novel types of packaging, such as active and intelligent packaging. The potential health benefits of edible films and coatings, as well as the biodegradability of films, are also discussed. Finally, this review addresses recent innovations in the edible films and coatings industry, including the use of nanotechnologies, aerogels, and probiotics, and provides future perspectives and the challenges that the sector is facing.

## 1. Introduction

Nowadays, there is a great variety of food packages, such as conventional glass, paperboard, aluminum, and plastics. In some cases, these could be replaced with more environmentally friendly packaging, such as biodegradable edible films and coatings made from biological sources. These films and coatings could be used on various products, such as meat, fish, milk, eggs, fruits, and vegetables. Edible films and coatings currently face important challenges. Initially, their goal is to extend the shelf life of perishable food products. More recently, there is a need to protect these products physically, chemically, and biologically. This is especially important for more complex products with additional nutritional properties, such as those that include probiotics and nutrients. The aim is to always use renewable raw materials and maintain environmental sustainability in line with the principles of the circular economy [1].

The first edible coating, wax, was made in 1992 and was used on fruit surfaces [2]. An edible film or coating is a material with a thickness of less than 0.3 mm, which is formed using a combination of biopolymers dispersed in aqueous media. Some authors use the terms “edible film” and “coating” interchangeably, but other authors distinguish between them according to the technique of incorporation in the food product. The edible coating is formed directly on the food, while the edible film is previously made and then adhered to the food product [1]. Some authors classify the edible coating when it has a thickness below 0.025 mm, whereas it is considered an edible film when the thickness is above 0.050 mm [2]. The term edible film may also be used in a different context, in the packaging, in this case, of food products [1].

Different methodologies can be used to produce edible films and coatings, with the main ones being casting, compression molding, and extrusion [3]. The techniques used to apply the edible film and coatings are knife coating, fluidized bed processing, panning, spraying, electrostatic spraying, dip coating, electro spinning, and layer-by-layer 3D food printing [2,3,4,5].

Films and coatings have principal functionalities that are fundamental for increasing the shelf lives of food products. They need to achieve protection against UV light and the transfer of compounds (e.g., solutes, water vapor, organic vapors, and gases) between the food and the surrounding atmosphere. They also need to act as a barrier against mechanical damage. The addition of functional/bioactive compounds, such as nutrients, antioxidants, and antimicrobials, against bacterial and fungal proliferation can be performed. The nutritional value can be enhanced with microorganisms that confer health benefits, such as probiotics. Aromatic compounds and flavors can also be added as enhancing agents. Additionally, the final package should be biodegradable and utilize biological materials [1,6].

The food packaging sector has changed, particularly to transform it into a sustainable system. Edible films appear as a new trend, with advantages and challenges such as antioxidant, antimicrobial, and nutritional properties, among others, but they essentially improve the shelf life of the product [7].

Edible films and coatings are derived from polymers and have an excellent potential to be incorporated with various additives in their matrix and released after storage. Active packaging extends the shelf life of food by capturing (scavenging) or diffusing (emitting) various compounds. Scavenging allows for the removal of undesirable compounds, such as oxygen, ethylene, carbon dioxide, and odors, from the internal packaging headspace. Emitters release substances that have a positive impact, such as antimicrobials, antioxidants, enzymes, nutraceuticals, and aromatic compounds [8]. This active packaging avoids the addition of antimicrobials directly to the food product and allows for controlled release during storage.

This review comprises a compilation of the most recent developments in edible film and coating production only using biological sources of components from a sustainability perspective and/or towards a circular economy approach; their main characteristics and functionalities; and their applications on fruits and fresh-cut fruits. It also focuses on the bio-based films used as packaging materials in novel systems, such as active and/or intelligent packaging. Finally, it approaches innovative technological advances, such as nanotechnology, and the use of probiotics in the formulations of edible coatings and films.

## 2. Biological Sources of the Compounds Used in Films and Coatings

The use of compounds of biological origin to make edible coatings and films is an issue of great relevance. To produce these materials from renewable sources, principally agro-industrial by-products and sea products such as algae, it is important to achieve results from a circular economy point of view. Table 1 lists the principal compounds used as bases in edible film and coating formulations and their respective sources, such as plants, macroalgae, animals, and microorganisms. Polysaccharides constitute the type of compounds most commonly used to produce edible films and coatings.

## 3. Edible Film and Coating Functionalities

Edible films and coatings can serve as functional bio-packages, pathogen inhibitors, and food preservatives. Additionally, the use of edible films and coatings can prevent non-microbial spoilage and physical defects in food. Factors such as rancidity, color loss, bleaching, fading of color due to enzymatic action, staleness associated with high-fat products or enzymatic changes in food, and dryness and limpness caused by enzymatic action can all affect the shelf lives of food products. To prevent these undesirable processes, base compounds with bioactivity can be incorporated into the formulation or bioactive compounds can be added to the food product or packaging (edible coatings or films) [1]. Various studies have identified and recognized several compounds with antioxidant, antimutagenic, anti-inflammatory, anticancer, apoptotic, and anti-cholesterol properties [1]. In this review, the term “biological activity” is used to describe the edible films/coatings that exhibited some form of bioactivity, such as antimicrobial and antioxidant activities, when applied/used in the foods.

Table 2, Table 3 and Table 4 present the compounds from biological sources that have recently (over the last three years) been used in coating and/or film formulations either individually or in combination, referring to their origin and respective food applications when applicable. Table 2 shows the edible coatings, and Table 3 outlines the films. Various compounds are used to produce coatings and films using the same formulation, as shown in Table 4. The functional/bioactive compounds that may also be incorporated into these edible coatings and/or films are also presented in these tables. The main results of the application on fruit and vegetable products are also presented, with most of the applications occurring in the postharvest field. The main results often include an extension of the shelf life, such as a reduction in water and texture losses, inhibition of microbial growth, prevention of oxidation, and maintenance of sensory quality.

From Table 2, Table 3 and Table 4, it is possible to conclude that the most commonly used compound was chitosan in edible coatings and starch in edible films (2021–2023).

The functionalities of some edible coating/film formulations are discussed below.

### 3.1. Bioactivity of the Edible Films and Coatings

Looking for sustainable alternatives, bioactive compounds from by-products may be used to convey their activities in the edible films and coatings, conferring them functions, such as antimicrobial, antioxidant, and anti-enzymatic properties, including therapeutic benefits for health, such as anticholesterolemic and other properties [3]. Through the inclusion of several additives, such as flavorings, vitamins, enzyme spices, dyes, and anti-browning agents in food, these ingredients contribute to its preservation to make, for example, fruit and vegetable supplies safer with extended shelf lives [145].

Often, edible films/coatings may be produced with more than one base compound, which is eventually incorporated with other compounds to enhance their specific functionalities.

#### 3.1.1. Antimicrobial Properties

Animals, plants, and microorganisms are sources of antimicrobial compounds, bacteriocins, enzymes, organic acids, and essential oils. It is possible to obtain essential oils and plant extracts from plants, enzymes (e.g., lysozyme and lactoperoxidase), polysaccharides (e.g., chitosan), and proteins (e.g., lactoferrin) from animals and from microorganisms (e.g., yeasts), or bioactive compounds (e.g., nisin and pediocin) from microbial sources [3].

Edible films/coatings based on polysaccharides, such as Arabic gum [30,146], guar gum [147], chitosan [148], fucoidan [65], pectin [149], and ulvan [150], on some proteins, such as milk protein derivatives/compounds, such as casein hydrolysate and casein phosphopeptides [151,152], and on lipids, such as candelilla wax [153], have revealed antimicrobial properties. In particular, fucoidan and ulvan have been shown to be antibacterial and antiviral [65,154,155]. In addition, when chitosan is used in edible films/coating formulations, it normally decreases the pH of the solution, which prevents microbial growth [118,148].

Some edible films/coatings may be incorporated with other compounds (such as extracts, protein/protein derivatives, essential oils, and phenols) to confer or enhance antimicrobial activity. For example, whey protein films incorporated with cinnamon essential oil (EO) showed high antibacterial activity against *E. coli* and *S. aureus* and an inhibitory effect on fungi [152]. Gelatine films incorporated with casein phosphopeptides showed good inhibition against *S. aureus* and *B. cereus* but not against *E. coli* [155]. Candelilla wax-based films with *Flourensia cernua* extracts showed antifungal activity against *B. cynerea*, *C. gloeosporioides*, and *F. oxysporum* [153]. Gelatine/guar gum (GG) bioactive films with green tea extracts (1%) were effective against *S. aureus* but not against *E. coli*, whereas GG/sodium caseinate films with cumin EO had high activity against *L. monocytogenes*, *S. aureus*, and *E. coli*, but not against *S. enteritidis* [147]. Carnauba wax coatings with grapefruit seed extract were effective against *M. fructicola* and *R. stolonifera* (completely inhibited) on mandarin surfaces [156]. Moreover, shellac coatings with tannic acid have antibacterial properties [79].

In addition, some edible films/coatings may be composed of two or more base compounds. For example, edible packaging based on milk proteins in combination with other compounds has revealed potential antimicrobial activity: the addition of casein hydrolysate (0.15–2%) to a whey protein concentrate edible coating increased its antimicrobial properties; composite films of casein and pectin, incorporated with clove EO, revealed high activity against *E. coli* [152]; conjugated (through a Maillard-type reaction) films/coatings with whey protein isolate (WPI) and bio fiber gum (BFG) and composite films/coatings with chitosan and BFG, both incorporated with 1% carvacrol, were effective against *Listeria*, *E. coli*, and native microorganisms in tomatoes and fresh-cut apples [142]. Blended edible films of pectin/tara gum with ellagitannins showed antimicrobial activity against *E. coli* and *S. aureus* [10]. Edible films of rennet casein with candelilla wax (1%) showed more than 25% higher antimicrobial activity than films with beeswax and activity similar to that of the films with carnauba wax [153].

As mentioned above, and illustrated with some examples, bioactive compounds may be incorporated into the films/coatings to confer or enhance the level of antimicrobial activity in fruit and vegetable applications. Some are present in essential oils, such as lemongrass EO [59], *Syzigium aromaticum*, and *Mentha spicata* EO [88], from plants, including Eos from agro-industrial by-products, such as *citrus sinensis* EO, in which the edible coating enhances the level of antibacterial activity [157], and lemon EO, in which the coating enhances the level of antifungal activity [74]. Other bioactive compounds may come from extracts, namely plant extracts and agro-industrial residues/wastes/by-products extracts. In the first group, there is ginger extract, whose coating was effective against *A. flavus* on walnuts [50], araçá extract, whose edible films inhibited the growth of *S. aureus* [130], and black tea extract [60]. In the second group, there is asparagus waste extract, whose coating is antifungal [24], mango peel extract, whose coating is antifungal and antibacterial [94], and pomegranate peel extract, whose film was effective against *E. coli* and *S. aureus* [112]. Many other different bioactive compounds have been used to try to increase the level of antimicrobial activity in fruits and vegetables, such as tea seed oil, whose coating was effective against *B. cinerea* [54], and thyme oil, whose coating was effective against *E. coli* and *S. aureus* [56], from plants, cardamom oil [34], and caraway oil [49] from by-products, and other compounds: curcumin [39], oleic acid [48], propolis [52], natamycin [63], bacteriocin from *Bacillus methylotrophicus* BM47 [158], citric acid [109], tannic acid [98], and xyloglucan [120]. Probiotics, such as *Lactococcus lactis*, together with cranberry extract [143], may also be incorporated into edible films/coatings to enhance their antibacterial activity.

Except for the authors who studied different edible film/coating formulations, as exemplified above [142,153], it is often not possible to compare these different formulations in terms of their antimicrobial activity because different authors may use different microbiological and quantification tests, e.g., counts in log CFU/cm^2^ [152]; inhibition zone or diameter in mm^2^ and mm, respectively [10,152]; and incidence rate in % [156].

#### 3.1.2. Antioxidant Properties

Oxidation is a major cause of food spoilage. Antioxidants are radical scavengers that trap free radicals, which delay and prevent oxidation, retarding both lipid oxidation and protein denaturation. Biological antioxidants are composed of simple phenols, phenolic acids, vitamins, tocopherols, carotenoids, flavonoids, and anthocyanins, with phenolic compounds being the most important group of antioxidant compounds [8,9]. These compounds may be used in packaging for food preservation; however, they should be low-cost, non-toxic, have high activity at low concentrations, and present good stability, not affecting the quality of the food. Such compounds should be chosen based on their molecular size, polarity, and release properties [8].

Some of the compounds described in Section 3.1.1 have also shown their antioxidant capacity. Edible films/coatings that have antioxidant properties are based on polysaccharides, such as alginate [17], Arabic gum [146], chitosan [148], fucoidan [65], guar gum [147], konjac gum [159], pectin [10,149], and ulvan [150], on some proteins, such as milk protein derivatives, like casein hydrolysate and casein phosphopeptides [151,152], and on lipids, like carnauba wax [156]. In spite of presenting good antioxidant properties, some compounds cannot be used alone as film packaging because they are too fragile, like the one with levan, which, to solve this problem, may be blended with gellan gum [125].

Some edible films/coatings may be incorporated with other compounds, normally with bioactivity, to confer or enhance their antioxidant activity. For example, a carrageenan edible coating containing lemon grass essential oil (EO) applied to strawberries showed a higher level of antioxidant activity (AA) than Arabic gum and xanthan gum edible coatings with the same EO [30]. Whey protein isolate with casein hydrolysate and an oolong tea coating prevented protein oxidation [152]. Some bioactive compounds mentioned above, which confer increased antimicrobial activity to edible films/coatings, also provide them with improved AA. For example, pectin coatings with lemongrass EO protected red guavas against lipid oxidation [59]. Chitosan/gelatine coatings with black tea extract [60], gellan gum probiotic films with cranberry extract and *Lactococcus lactis* [143], pectin coatings with bacteriocin from *Bacillus methylotrophicus* BM47 [158], xanthan gum coatings with citric acid [160], and alginate/gelatine/Ag films with tannic acid [98] increased the AA of minimally processed papayas, fresh-cut apples and potatoes, blackberries, fresh-cut lotus roots, and tangerines, respectively.

As for the antimicrobial activity, it is often difficult to compare the different formulations in terms of their antioxidant activity because different authors may use different methods of determination, e.g., DPPH radical scavenging activity in mg Trolox equivalents/g [147], or radical cation scavenging rate in % [10]; ABTS [112]; and ferric ion reducing antioxidant power [147].

#### 3.1.3. Anti-Enzymatic Capacity

Some authors have reported that the use of coatings and films to increase the shelf lives of products involves the modulation of enzymatic activity. For example, a coating made with plant extracts may be used to reduce the activity of polyphenol oxidase (PPO) and peroxidase (POD) [2,161,162]. Another way of acting upon enzymes to increase the shelf life is through the promotion of the activity of antioxidant enzymes, such as glutathione peroxidase, ascorbate peroxidase, and guaiacol peroxidase, using plant-based extracts [161].

Some specific films incorporated with bioactive compounds may exhibit anti-enzymatic properties. Carboxymethyl cellulose-based coatings with *Morus alba* root extract controlled PPO activity [35], whereas bacterial cellulose coatings with chia seed mucilage, a by-product, controlled PPO and POD activities [33]. Chitosan/gelatine layer-by-layer coatings incorporated with lemongrass EO and β-cyclodextrin enhanced the level of catalase activity [59], whereas xanthan gum coatings with citric acid decreased enzymatic browning [160].

### 3.2. Physical Properties of Edible Films and Coatings

Some physical properties of edible films and coatings, such as resistance to water, oils, and fats, are also critical in the selection of a edible film/coating for a given food application. Normally, a film resistant to water, in most cases hydrophobic, is sought for food applications. For example, alginate [17] and pectin [10,149] films and coatings are not resistant to water, which is essentially related to their permeability to water vapor and gases and their solubility in water. Nevertheless, alginate coatings offer a good form of resistance to oils and fats, and they are also a good barrier to oxygen [17], which may constitute positive characteristics of an edible film/coating. Many other compounds, such as agar [16], Arabic gum [30,146], carboxymethyl cellulose [163], dextran [164], and guar gum [147], all polysaccharides, and some proteins, such as casein [152] and soy protein [116], were found to be soluble in water and/or hydrophilic; therefore, they are not good candidates to constitute, alone, an edible film/coating resistant to water. On the other hand, some polysaccharides, such as konjac gum/curdlan [64], hydoxymethyl cellulose/curdlan [113], and gellan gum [125,126,165], some proteins, such as corn zein [93] and gelatine [166], and some lipids, such as candelilla wax [153] and carnauba wax [156], produce edible films/coatings with low or no permeability to/solubility in water, some of which are hydrophobic. Nevertheless, proteins have been proven to present a higher moisture resistance and better mechanical properties in comparison to polysaccharides films. In addition, they improve nutritional values and sensory properties [106] and provide health benefits to consumers [151,152].

To improve their physical properties, such as mechanical resistance, some edible films/coatings may be composed of two or more base compounds. For example, agar/cellulose films/coatings with gelatine, gellan gum, k-carrageenan, or tamarind gum have shown increased tensile strength [16]. Whey protein–kefiran films presented a good level of mechanical resistance [167]. The mechanical resistance, water resistance, and other physical properties of soy protein isolate films can be reinforced with silylated nanocellulose [116]. Wheat proteins with alginate films offer good mechanical resistance and reduced water solubility [106]. Edible films with high methoxyl pectin and pea protein isolates become thicker, stronger, and stiffer as the concentrations of these compounds increase [111].

Permeability to oxygen is also an important parameter because, to prevent food oxidation, the edible film/coating should be impermeable to this gas. Normally, the impermeability to O_2_ goes hand in hand with the impermeability to other gases, such as CO_2_. Some polysaccharides, including gellan gum [125,126,165], kefiran [167], pullulan [141], starch [168], and tara gum [169], and proteins, including corn zein [93], gluten [127], wheat protein [106], and whey protein [167,170], can be used to create films that are a good barrier to O_2_, thus protecting foods and food ingredients from oxidation. In particular, pullulan is also impermeable to oil, and it is transparent and odorless [171]. However, gluten-based films do not have a good level of mechanical strength, and other compounds, such as guar gum and Persian gum, may be used to solve this problem [127]. On the other hand, the films and coatings made from chitosan are permeable to gases, namely O_2_ and water vapor, despite their excellent mechanical properties [148].

Some edible films/coatings based on specific compounds, such as gellan gum, levan/gellan gum, ulvan, and casein, showed good thermal stability [125,150,151,152,165], which is also a positive aspect to consider for food applications. Pectin films reinforced with spent coffee grounds, which are wastes from coffee industries, also presented good thermal stability [172].

Some studies have demonstrated that the use of plant extracts affects the physical characteristics of coatings and films. For example, it may decrease their thermal stability, elongation at break, and tensile strength, and improve their scavenging activity, opacity, solubility, and permeability to water vapor [173]. Chitosan-based ternary blend films with gelatine and cinnamon EO showed high elongation at break but low tensile strength [174]. Alginate films with *Vitis vinifera* leaf extract exhibited high tensile strength [100]. The incorporation of coconut oil, tannic acid, and sunflower oil in agar/Arabic gum/konjac gum [96], alginate [98], and Persian gum [128] films, respectively, decreased the levels of water vapor permeability and solubility in water and/or water swelling. Some probiotics enhance the hydrophobicity and/or the barrier against water vapor of carrier films, such as *Bacillus coagulans* in alginate-based films [99]. Finally, the transparency of the edible film/coating may also be an important parameter for deciding their usage. Gellan gum [125,126,165], gelatine [166], and pullulan [171] are some examples of base compounds that present a high level of transparency.

Several studies have been performed on coatings and films to protect foods from UV-B radiation, and it has been concluded that the optical properties are affected by the coating’s thickness, the base compounds, and the bioactive compounds incorporated. López-Ortiz et al., 2021 [175], concluded that xanthan gum, Arabic gum, and guar gum alone do not have anti-UV radiation capacity, but when fenugreek seed (*Trigonella foenum-graecum*) extract was added to the edible coatings, they showed high UV absorbance and practically zero transmittance, making them excellent solar filter coatings. Xanthan gum is mainly used as a stabilizer, thickener, or emulsifier, and Arabic gum may be used in encapsulation and emulsification [30,146]. Another study by Aziz et al., 2022 [18], using alginate and zinc oxide nanoparticles and aloe vera extract, showed excellent UV shielding compared with alginate films alone. Another author proposed a film with gelatine and esculin, resulting in increased transparency values when the amount of esculin was increased in the film. This compound could also improve the UV barrier properties of gelatine films [176].

### 3.3. Other Properties

In the formulation of intelligent packaging, the use of pH-sensitive bioactive compounds is common. An example of the use of these compounds is anthocyanins, a class of phenolic compounds that change their color from blue, purple, and red when the environmental pH is modified. The films and coatings that are rich in anthocyanins provide antioxidant and antimicrobial activities to the film, and they may provide colorimetric information for monitoring the freshness of the food product [9]. In addition to anthocyanins, other compounds, such as betalains and curcumin, can also change colour with the pH [9,14,102,177]. The potential of anthocyanins from *Brassica* sp. and *Clitoria* sp. edible plants in a carrageenan-based film was evaluated as a colorimetric pH sensor, which is an indicator of food freshness during storage [178]. Another example is the use of anthocyanins for the same purpose in alginate/beeswax films [102].

### 3.4. Pitfalls of the Bioactives Present in Edible Films and Coatings

The limitations of the use of bioactive compounds from biological sources are their easy degradation, low water solubility, low bioavailability, and undesirable taste. In addition, food constituents, such as fat, minerals, vitamins, salts, and proteins, may interact with some bioactive compounds and modify their mechanism of action. The use of technologies, such as microencapsulation and nanoencapsulation, may be recommended in order to both protect bioactive compounds from degradation and enhance their solubility and bioavailability while also masking any potential undesirable tastes. In fact, if some essential oils are not encapsulated, they may alter the colour or sensory characteristics of foods. In addition, these technologies allow for the slow release of the relevant functional/bioactive compounds.

Some essential oils are prohibited because of their cytotoxic effects, toxicological reasons, or allergenicity. Therefore, only GRAS essential oils should be used, with no toxic or allergenic effects.

In addition, the storage conditions of temperature and relative humidity may also have a significant impact on the properties and release of bioactives from edible films [3]. Therefore, it seems of the utmost importance to evaluate and select adequate storage conditions to retard the release of bioactives while maintaining their characteristics.

Another important issue is an economic one, as some bioactive compounds may not be easy to extract, and the extraction methodology may become expensive, e.g., ethanolic extraction assisted or not with ultrasound or microwaves and supercritical fluid extraction. Extracts rich in bioactives are recommended to be obtained using green methodologies of extraction to avoid the use of toxic solvents and high-cost energy methodologies.

Edible antimicrobial packaging is essential to complement food safety by preventing the development of resistant microorganism strains. It is a challenge for science to identify the antimicrobial compounds that do not create resistant strains. In addition, if the targeted pathogen has a very short lag phase, the biopolymer slowly releasing antimicrobial compounds will be ineffective in controlling its growth. Moreover, if the antimicrobial incorporated into the edible packaging is not released from the edible film, it may not be effective [3]. Therefore, there is a need for further in vitro and in vivo studies on the release of these compounds.

From Table 2, Table 3 and Table 4, it can be concluded that research in this area tended to increase after the COVID-19 pandemic.

## 4. Health Effects of Edible Films and Coatings

There is limited research in the literature on the effects of edible films and coatings on human health, specifically on fruits and vegetables coated with these films and coatings. For instance, Ajayi et al., 2023 [179], conducted a study on the use of chitosan and chitin as coatings on fruits and vegetables to fight inflammation in metabesity. Metabesity refers to metabolic diseases, such as obesity, diabetes, metabolic syndrome, cardiovascular disease, neurodegenerative disorders, accelerated aging, and cancer. However, some studies have focused on the base compounds of edible films and coatings, as well as the inclusion of bioactive compounds in these formulations. One example is fucoidan, which possesses antibacterial, antiviral, and antioxidant properties (already discussed), and has also been reported to provide health benefits, such as anticancer, immunoregulatory, anti-thrombotic, and anti-inflammatory effects [155]. Although the health properties of fucoidan-based edible coatings have not been specifically tested, they do constitute potential functionalities. The antioxidant activities of fucoidan can vary depending on various factors, such as concentration, molecular weight, and degree of sulphation [154]. Additionally, polyphenols have demonstrated anticancer properties [180].

A more sensitive issue is research related to viruses, which can also be responsible for serious health problems. Norovirus (NoV) is a human enteric pathogen that can cause acute gastroenteritis and may contaminate fresh/fresh-cut fruits and vegetables. Leite et al., 2023 [181], reported that several compounds have shown activity against various viruses with relevance to human health, such as persimmon extract against NoV, carvacrol against murine virus, curcumin against hepatitis B virus, and tannic acid against hepatitis C virus. Therefore, when these compounds are incorporated in edible films and coatings, as illustrated in the present manuscript, they will potentially present similar activities and guarantee food safety and health benefits. Cerqueira et al., 2023 [182], showed that sodium alginate-based films with gallic acid, geraniol, or green tea extract had strong in vitro antiviral activities against SARS-CoV-2. Geraniol and green tea extract were effective at lower concentrations (0.313%) in the respective films than gallic acid (1.25%). In addition, films with gallic acid lost their activity after the second week of storage, whereas films with geraniol and green tea extract showed a decrease in activity after four weeks. This suggests that these edible films may be applied to fruit and vegetable products of relatively short shelf lives to successfully protect them against SARS-CoV-2, which could contribute to reducing the spread of this virus through the food chain. In fact, these three compounds showed several biological properties: geraniol—antioxidant, anti-inflammatory, and antimicrobial activities; green tea—antioxidant, anticarcinogenic, anti-inflammatory, and antimicrobial (bactericidal and virucidal) properties against various food-borne pathogens; and gallic acid—anti-inflammatory, anti-diabetic, cardioprotective, anticancer, and hepatoprotective activities.

Another type of study is the in vitro digestion simulation of coated fruit and vegetable products or of the coating ingredients, which has been little addressed. For example, agave polysaccharides, which are important agro-industrial wastes, are promising sources of prebiotic polymers with potential beneficial effects on intestinal health [183]. The bioaccessibility of phenolic compounds in green tea extract incorporated in agar films was studied during simulated digestion in the upper gastrointestinal tract using a dynamic gastric model and a static duodenal model. The recovery of the tea compounds incorporated in the agar film mainly occurred in the stomach (50–80%), whereas little or no additional recovery was observed in the duodenum. Furthermore, the bioaccessibility of green tea flavonols was reduced in the presence of gelatine, which was used to simulate the presence of proteins in the stomach [184].

The consumption of probiotics [185] also brings health benefits, such as maintaining inflammatory control and the microbiota (immune system), regulating the growth of pathogenic microorganisms, such as *Clostridium difficile* and *Helicobacter pylori*, modulating brain functions (nervous system), and helping to control dermatitis and allergies (skin). For example, lactic acid bacteria (LAB) from *Lactobacillus* play an important role in preventing the deterioration of the microbiota and inhibiting pathogenic microorganisms (bacterial pathogens and fungal agents) in the oral cavity and colon [186]. Soukoulis et al., 2014 [187], evaluated the survival of *Lactobacillus rhamnosus* in an alginate/whey protein matrix by testing some prebiotic fibers. LAB stability was maintained for seven days at 25 °C. Wong et al., 2021 [188] developed a bilayer edible coating with CMC containing *L. plantarum* and zein on fresh-cut apples and observed that the probiotic bacteria were stable for 7 days at 4 °C (>6 log CFU/g), but only manifested a decrease of 2.24 log CFU/g during simulated digestion. In a different study, several polysaccharides, low-methoxylated pectin, k-carrageenan, sodium alginate, and pullulan films exhibited good oxygen barrier properties for protecting extremely oxygen-sensitive probiotics in contact with a simulated gastrointestinal tract; sodium alginate exhibited the best oxygen barrier properties and release profile [189]. A symbiotic film was developed from cassava starch (base compound), inulin (prebiotic), and *L. casei* (probiotic) bacteria. The viability of *L. casei* at 10 °C and 25 °C was lower after storage and higher temperatures, and its viability under the simulated gastric conditions was lower on cassava starch films than when inulin was included. Inulin appeared to have a protective effect on the probiotic bacteria because cassava starch is known for its low resistance to acid hydrolysis, leaving *L. casei* unprotected in gastric media [190]. More studies are certainly required on this area.

## 5. Biodegradability of the Films

Baghi et al., 2021 [8], described biodegradation according to the European standard EN13432 [191]. Various tests have been used to evaluate the biodegradation of films. Most tests are performed using soil. Vargas et al., 2023 [192], observed that gelatine-based films lose their integrity after 12 days in the soil. Rohadi et al., 2023 [193], used a test in which the film structure was broken down by microorganisms in the ecosystem soil and observed that cellulose, gelatine, and chitosan films suffered a high weight loss of 30% and 70% in 7 days and 28 days, respectively. Thakwani et al., 2023 [194], determined that starch films could be degraded in composting soil in 5–7 weeks at 25–30 °C, while another author, Frangopoulos et al., 2023 [195], found that the same compound-based films disintegrated after only 10 days of thermophilic incubation under their simulated composting conditions. Kesari et al., 2022 [196], confirmed a 70–85% degradation of cellulose films within 60 days, whereas Zhao et al., 2019 [197], stated that they could be broken down 100% in soil in half of this time. Gan et al., 2022 [141], concluded that levan, pullulan, and chitosan had good degradability in soil after only seven days. Zhou et al., 2022 [198], tested the biodegradability of carrageenan at approximately the same time. According to Bandyopadhyay et al., 2019 [199], cellulose and guar gum film degradation was 80% after 28 days in vermicompost. Capar et al., 2023 [100], verified the 90% degradation of alginate films over 30 days in soil.

A new method, by area loss assessment through digital image analysis, was recently proposed to assess the biodegradability of films by Azevedo, et al., 2023 [200]. Alginate–gelatine and alginate–chitosan films suffered area losses ranging from 28% (with bioactive compounds incorporated in the film) to 90% after 30 days under indoor soil conditions.

Heredia-Guerrero et al., 2023 [201], determined the biodegradability of a chitin-based film (chitin deacetylation originating from chitosan) by measuring the biological oxygen demand (BOD) in seawater. Using this method, they observed the deterioration of the film’s surface through the actions of microorganisms in seawater. They observed that the increase in glycerol concentration favored the degradation of the film. The biodegradability, assessed through BOD and weight loss, of this chitin-based film was similar to that of pectin (Moreno et al., 2021) [202] and cellulose films [203], using the same evaluation methodology. Using a different test of aerobic biodegradability with activated sludge sampled from a municipal wastewater treatment station, casein films were found to be 80% degraded in approximately 20 days [204].

Lipids are hydrophobic and difficult to biodegrade. In fact, their hydrophobicity is one reason why they are mixed with polysaccharides and proteins—to increase the water barrier properties of these compounds. In fact, the barrier properties are inversely proportional to the degree of biodegradability [205]. Recent studies have attempted to solve this problem. For example, Wang et al., 2023 [91], proposed a hydrophobic and biodegradable coating using polylactic acid and carnauba wax.

In summary, films based on some compounds approached in the present study seem to present quite good biodegradability, sometimes in a short amount of time (one week), and some reach a high percentage of biodegradation from 70% to 100% within approximately 30 days, such as cellulose, which has been most studied, and alginate.

## 6. Innovations in the Edible Film and Coating Industry

Recent innovations have turned edible films and coatings into functional bio-packages, such as pathogen inhibitors and food preservatives, which help the food industry offer fresh, pleasant, good-quality food with beneficial health properties to consumers. Here, the bioactive compounds from biological sources, particularly those from by-products, may have a fundamental role. It is important to match their biological activity with the industry needs in active and intelligent packages [206,207,208].

Nanotechnology can help mitigate the problems of degradation, low solubility, bioavailability, and undesirable taste of specific compounds, and it has been used in recent studies in the field of edible films and coatings. Encapsulation is a promising technology that enhances physical, chemical, and thermal stability. It also allows for controlled release and targeted delivery [209]. Recent studies have focused on matching bioactive compounds with shell materials used in encapsulation [8]. Various biopolymers have been used to encapsulate food ingredients, taking into account the characteristics of nano- and micro-encapsulates, as applied in the encapsulation of food ingredients [210]. These biopolymers include polysaccharides, such as xanthan gum, modified starches, maltodextrins, alginates, pectin, carrageenan, cellulose derivatives, chitosan, and cyclodextrins. Additionally, fats and waxes, like hydrogenated vegetable oils, beeswax, lecithin, medium-chain triglycerides, and glyceryl behenate, as well as proteins, such as gelatine, whey protein, sodium caseinate, soy protein, gluten, caseins, zein, and silk fibroin, have also been utilized for encapsulation purposes. For example, cauliflower-derived plasma membrane vesicles were used to nanoencapsulate the extracts of Bimi^®^ edible parts. These vesicles showed, during in vitro gastrointestinal digestion, a higher capacity for the retention of 3,3-diindolylmethane, indole-3-carbinol, and sulforaphane, which are isothiocyanates present in the extracts and may be used as food ingredients [211]. Nanoemulsions possess two crucial properties that facilitate the encapsulation and release of essential oils from edible coatings and films into food products. An example is the use of essential oils with rather hydrophobic properties (edible films formed using polysaccharides are rather hydrophilic), which are dispersed to form an emulsion in a way to improve the compatibility between essential oils and polysaccharides before their incorporation into film-forming suspensions [212]. Biological materials are generally inexpensive, readily available, often non-toxic, and can be used in food formulations. When designing the encapsulation process, certain factors should be taken into account. These include the size of the capsule, the final physical state, their stability, and the physical conditions required for release [209,210].

There are recent promising technologies, e.g., allowing the application of thin edible coatings through spraying and providing a uniform and efficient method of coverage (sprayable coatings), and electrospinning has been explored for creating nanofiber-based films with unique properties, such as high surface area and porosity [4].

The use of aerogels made of polysaccharides in active food packaging is also a recent development. Aerogels are a lightweight and highly porous solid material with a large surface area, high mechanical stiffness, and low thermal conductivity. These aerogels have been studied because of their ability to easily incorporate bioactive components, nutrients, and drugs. Researchers have explored the use of polysaccharides to create these bioaerogels because of their biodegradability, biocompatibility, and edibility, and potentially to replace synthetic materials. Polysaccharide aerogels have a low level of thermal conductivity, making them ideal for insulating hot or chilled food and beverages. They can also act as carriers for nutrients and bioactive compounds, preventing microbiological contamination in foods and absorbing water vapor and oxygen [11].

Another innovative approach involves using certain microorganisms in edible films and coatings to inhibit the growth of pathogenic microorganisms. Probiotics are microorganisms with specific health benefits, as discussed in Section 4.

## 7. Future Perspectives

**Figure 1:**
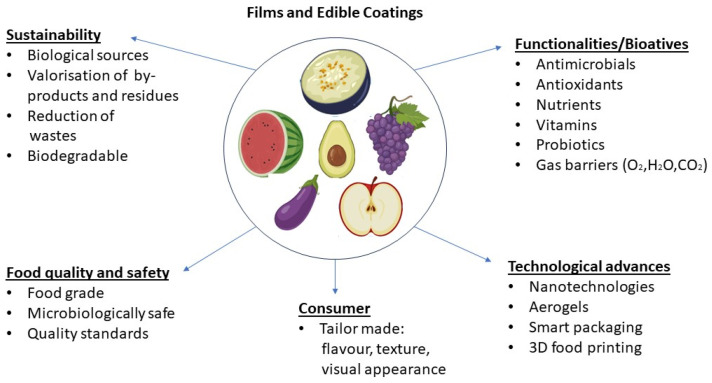
Future trends for edible films and coatings.

Future developments in edible films and coatings in the food industry will certainly be driven through a combination of consumers’ preferences, sustainability concerns, and technological advancements while addressing food safety and waste reduction (Figure 1).

In agreement with the core of this review paper, a strong emphasis will be placed on the development of sustainable edible films and coatings made from biological ingredients. These include polymers from biological sources, including plants, animals, algae, and microorganisms, that are biodegradable and, therefore, environmentally friendly. Examples of these compounds, such as chitosan, cellulose, starch, and alginate, were discussed in the present work. In addition, sustainable packaging will continue to be a significant trend, and films made from biological sources can contribute to eco-friendly packaging solutions by reducing the need for traditional plastic packaging. This will also contribute to reducing packaging waste. Edible coatings will also continue to evolve as a means of reducing food waste because they can extend the shelf lives of fresh/fresh-cut fruits and vegetables and minimize spoilage, as demonstrated in the present review, through the results obtained by so many researchers in recent years. Among the biological sources of ingredients for edible film and coating production, the by-products, residues, and wastes from the agro-food industries present a high potential. Examples of these are also given in this review.

The trend of enriching edible coatings with functional/bioactive ingredients, such as antioxidants, vitamins, and probiotics, to enhance the nutritional value of coated food products will continue. This brings along the need for more research studies on the simulation of digestion of such coated foods in the gut to determine if they do constitute functional foods. From the consumer’s perspective, the use of edible coatings to modify the texture and flavor of foods will continue to be explored. Customizable edible coatings may become more common, allowing for the consumers to tailor the appearance or flavor and enhancing the visual appeal of food products, making them more aesthetically pleasing for consumers, without neglecting the food safety issue. Here again, edible coatings can be incorporated with antimicrobial compounds, inhibiting the growth of harmful microorganisms on the food surface of the fruit or vegetable, as illustrated in the present work. Nanotechnologies, as delivery systems granting protection to the bioactivity of compounds, continue to be promising in this area through the creation of ultra-thin films and coatings.

Nevertheless, all ingredients used in edible film and coating formulations should be food-grade, and manufacturers will have to comply with food safety and quality standards.

In addition, the use of intelligent packaging may include features such as temperature monitoring, freshness indicators, providing real-time information on the quality or safety of the packaged product, and QR codes for traceability. Finally, 3D food printing and edible inkjet printing will become more sophisticated, enabling visually appealing edible designs on food products. The integration of printed sensors onto packaging materials will also allow for real-time monitoring of food freshness and safety.

## 8. Conclusions

Intensive research on edible films and coatings has recently been developed from a sustainability perspective. Different biological sources of matrix biopolymers for these materials have been used, mainly plants, animals, algae, and microorganisms, including by-products, residues, and wastes from the agro-food industries. Plant-based polysaccharides are the most commonly used biopolymers. Nevertheless, chitosan, derived from an animal source, has been the most studied compound over the last three years. Films based on this compound have the advantage of being biodegradable, which is an environmental concern regarding film packaging disposal. Other polysaccharide-based films, such as those based on cellulose and alginate, exhibit a good level of biodegradability, as well as films based on certain proteins, such as gelatine and casein. Lipid-based films, on the contrary, are difficult to biodegrade, such as those based on waxes, like candelilla wax and beeswax. Nevertheless, these may be selected for film packaging because of their high resistance to water. Polysaccharides normally do not exceed 3–5% in the film/coating base solution, with the exceptions being Arabic gum, guar gum, cellulose, tragacanth, tamarind gum, pectin, and starch. Proteins and lipids may be present in higher concentrations, including zein (up to 20%) and beeswax (up to 40%).

Different compounds/substances/microorganisms have also been used to enrich the main polymeric matrix to confer or enhance its properties, including bioactivity or functionality and nutritional value, such as antimicrobial and antioxidant activities and physical properties. The addition of living bacteria, probiotics, has also been investigated to produce potentially beneficial effects on human health, and some in vitro digestion studies in the gut have been performed. Nanoencapsulation and aerogels of different compounds are proposed to protect them, maintain their bioactivity/functionality, and potentially guarantee a slow release after consumption.

All of these edible film/coating formulations were successfully applied to several fruit and vegetable products, mostly in the postharvest field, either on their surfaces or as packaging, resulting, for example, in shelf life extension, inhibition of microbial growth, prevention of oxidation, and maintenance of sensory quality. Active packaging and intelligent packaging are also important applications, such as quality indicators.

Further research on several compounds from renewable sources to produce edible coatings and films is recommended. In addition, some biological resources, such as microalgae, have not been extensively explored as potential sources. More studies are also required on the in vitro simulation of digestion in the gut of coated fruit and vegetable products to assess their potential benefits to human health. From the consumer’s point of view, more sensory studies are recommended.

## Figures and Tables

**Table 1:** Compounds used as a base for edible films and coatings and their principal biological sources [6,8,9,10,11,12,13,14,15,16].

Biological Source				
Compound Class	Plant	Macroalga	Animal	Microorganism
Polysaccharide	Arabic gum Basil seed gum Cellulose and derivatives Corn fibre gum Dextran Flaxseed gum Guar gum Kefiran Konjac gum Maltodextrin Pectin Persian gum Quince seed gum Starch Tamarind gum Tara gum Tragacanth gum Pullulan	Alginate (brown alga) Agar (red alga) Carrageenan (red alga) Fucoidan (brown alga) Laminarin (brown alga) Ulvan (green alga)	Chitosan	Alginate (Bacteria: *Pseudomonas aeruginosa*) Cellulose and derivatives Chitosan (fungi) Curdlan Cyclodextrin Gellan gum Levan Pullulan (*Aureobasidium pullulans*) Xanthan gum
Protein	Gluten Quinoa protein Wheat protein Zein		Casein Gelatine Soy protein Whey protein	
Lipid	Candelilla wax Carnauba wax Glyceride		Beeswax Shellac Glyceride	
Other polymers				Polyhydroxyalkanoate (PHA) Poly(β-hydroxybutyrate) (PHB) Poly(3-hydroxybutyrate-co-3-hydroxyvalerate) (PHBV) Polylactic acid

**Table 2:** Recent bio-based edible coatings used in fruit and vegetable food applications and their biological sources (2021–2023, ScienceDirect, Google Scholar databases).

Coating Base, Base Solution Concentration	Biological Source	Incorporated Ingredient/s	Food Application: Food Product, Main Results	Reference/s
Alginate, 1–3%	Macroalgae (*Macrocystis pyrifera*, Kelp)	-	Rose apple cv. *Tabtimchan*, retarded CI.	[17]
		Aloe vera and frankincense oil; aloe vera and garlic oil; aloe vera	Green capsicum, excellent inhibition of bacteria and fungi; tomato, mechanical, thermal, and antimicrobial properties, UV shielding.	[18,19,20]
		Limonene; loquat leaf extract	Blackberry, lowered microbial growth; *Nanfeng* tangerine, extended SL.	[21,22]
Alginate, 1%/CMC, 1%/starch, 1%	Macroalgae/plant	Grapefruit seed extract	Green chili, enhanced the SL by 25 d.	[23]
Alginate, 1%/cellulose (hydroxyethyl), 0.5%		Asparagus waste extract	Strawberry fruit, activity against *Penicillium italicumy*, reduced color changes and WL, maintained TPC and flavonoid content, and extended the SL.	[24]
Alginate, 0.2–0.5%/chitosan, 0.2–0.5%	Macroalgae/animal	-	Japanese pear fruit, extended the SL.	[25]
Arabic gum, 2–10%	Plant	Bergamot pomace extract or bergamot EO; *Zataria multiflora* Boiss EO	Strawberry, low decay rates, good acceptability by consumers, and retention of ascorbic acid for 14 d; pistachio, high free fatty acid and peroxide values.	[26,27]
Arabic gum, 10%/CMC, 0.5%		-	Tomato, extension of ripening phase, delaying senescence, and increasing acceptability for longer time.	[28]
Carrageenan (k), 0.2%/chitosan, 0.75%	Macroalgae/animal	-	Dragon fruit, maintained freshness and bract color, retained chlorophyll content and fruit eating quality.	[29]
Carrageenan, 0.5%/Arabic gum, 3%/xanthan gum, 0.1%	Macroalgae/plant/bacterial	Lemon grass EO	Strawberry, inhibited psychrophilic bacteria, yeast, and mold, retained quality up to 12 d under refrigeration.	[30]
Casein, 0.5%	Animal	Gliadin nanoparticles; methyl jasmonate	Cherry tomato, controlled black rot, alleviated CI during long-term cold storage.	[31,32]
Cellulose, 0.6–0.8%	Bacterial (*Gluconacetobacter xylin)*	Chia seed mucilage	Strawberry, controlled PPO and POD.	[33]
Cellulose (CMC), 1–1.5%	Plant	- Cardamom oil; *Morus alba* root extract	Strawberry, increased the SL better than pectin, tragacanth and persian gums (efficiency in this order); tomato, prevented microbial spoilage; banana, controlled colour, PPO, and BI.	[13,34,35]
Cellulose (CMC), 2–10%/pectin, 2–10%	Plant (pectin from banana peel)	-	Tomato, increased SL in cold storage; fruits and vegetables, prevented microbial decay and enzymatic/biochemical, physical/textural changes.	[36,37]
Cellulose/chitin/chitosan (1:1:1)	Plant (*Miscanthus* *floridulus* straw)/animal (crab shells)		Strawberry, decreased WL and color changes.	[38]
Cellulose, 1%/chitosan, 1.5%		Curcumin	Kiwifruit, reduced WL, firmness loss, and microbial growth for 10 d at 10 °C.	[39]
Cellulose (hydroxypropyl methyl), 1%/carnauba wax, 9–18%	Plant	Ginger EO	Papaya, reduced WL, color development, and slowed ripening.	[40]
Cellulose (hydroxypropyl methyl), 5%/beeswax, 10–40%	Plant/animal	-	Mango, increased the SL by 6 d.	[41]
Cellulose (nanofiber), 0.8%/zein, 0.8%	Plant (softwood Kraft pulp)	Beeswax or camellia wax emulsion	n.a.	[42]
Cellulose, 4–16%/pectin, 1%	Bacterial/plant (citrus peel)	Blackberry pomace	n.a.	[43]
	Plant (citrus fruit)	*p*-coumaric acid	Fresh-cut peach, inhibited the browning process within 8 h.	[44]
Cellulose, 0.5%/starch, 4–5%		Basil EO	Mandarin, increased the SL in 12 d at 25 °C.	[45]
Cellulose, 0.5–1.5%/carnauba wax, 0.5–1.5%		-	Pomegranate, extended the SL to 150 d.	[46]
Chitosan, 0.05–2%	Animal (crab shells; shells of *Litopenaeus vannamei;* crustacean shells)	Caraway oil; canola oil, ginger extract; grapeseed EO, sea buckthorn EO; mixed plant extract of moringa + eucalyptus + marigold; oleic acid; pomelo extract; propolis extract; tea polyphenols; tea seed oil; thymol; thyme oil; *Torreya grandis* seed EO	Banana: reduced WL, firmness loss, TSS, and TA, maintained color, increased AA, and inhibited microbial growth. Walnuts, reduced *A. flavus* incidence and spores. Organic strawberries and apples, SL extension. Pitaya, prevented fungi for 15 d and maintained quality parameters. Lychee, inhibited fungal decay and improved storability. Fig, aflatoxin production < 20 ppb and acceptable sensory quality. Broccoli, improved sensory quality and nutraceutical value. Japanese pear, activity against B. *cinerea*. Grape, improved firmness, AA, anthocyanin, and sensory attributes, extending the SL. Mango, controlled anthracnose better than fungicides. Loquat, reduced WL and decay index and increased TSSs and ascorbic acid.	[47,48,49,50,51,52,53,54,55,56,57]
Chitosan, 0.5–2.5%/gelatine, 1–5%		- Black tea extract; lemongrass EO; and β-cyclodextrin	Red guava, delayed ripening, reduced WL and lipid oxidation, maintained color and firmness, and preserved for at least 8 d; papaya, suppressed microbial growth, increased the pH, TSSs, TA, AA, and total carotenoids during storage due to substances in the edible layer; cherry tomato, very effective against fungi for 20 d.	[58,59,60]
Chitosan, 1–1.5%/guar gum, 0.3–25%	Animal/plant (citrus peel)	-	Kinnow fruits, extended the SL up to 25 d at room storage.	[61]
Chitosan, 1%/Arabic gum, 10%		*Cleistocalyx operculatus* extracts and natamycin	Banana, 21 d of storage at room conditions; black Périgord truffles, can affect volatile organic compounds and bacteria implicated in preservation.	[62,63]
Curdlan, 1%/konjac gum, 1%	Bacterial/plant	-	Cherry tomato, reduced WL, decay loss, firmness loss, TSSs, total acid, and volatile compound contents.	[64]
Fucoidan, 1–5%	Algae		Mango fruit, extended the SL.	[65]
Gelatine, 1–2%	Animal (fish skin)	- Almond gum	Peanut, binary system of lignin and gelatine favored quality control; tomato, did not affect the pH and colour indices, delayed changes in firmness, lycopene content, WL, and decay.	[66,67]
Gelatine, 14.5%/starch, 9.20%	Animal (poultry waste)/plant (lotus)/plant (cassava)	-	Cherry tomato, maintained firmness and the pH and reduced WL during 15 d of storage.	[68]
			Banana, delayed respiratory peak by 4 d.	[69]
Guar gum, 1–2.5%	Plant	Castor oil	Mango, increased TPCs and AA and promoted SL extension.	[70]
Guar gum, 2.5%/starch, 2.5%		-	Cut apple, storage quality in terms of microbial growth, pH, color, and WL.	[71]
Maltodextrin, 4%/pectin, 6%		Sodium chloride	Starfruit, extended the SL and maintained physicochemical characteristics for 14 d.	[72]
Pectin, 0.5–3.5%		Carvacrol/2-hydroxypropyl-β-cyclodextrin	Strawberry, improved the SL, reduced WL, decay, and preserved nutritional ingredients; n.a.	[13,73]
	Plant (orange peels)	Lemon EO or Lemon EO and reuterin from *Lactobacillus reuteri*	Strawberry, avoid fungal spoilage without quality reduction.	[74]
Pectin, 1.5–3%/beeswax, 10%	Plant/animal	*Artocarpus heterophyllus* leaf extract	Tomato, controlled *Alternaria* spp., improved the SL.	[75]
Persian gum, 4%	Plant	-	Strawberry, reduced WL and decay, preserved nutritional ingredients at 4 °C.	[13]
Shellac, 1–20%	Animal	- Juglone and tannic acid	n.a. Lime, delayed color changes, reduced chlorophyll degradation, affected TA reduction, and enhanced the accumulation of total ascorbic acid and hydrogen peroxide; Wichita pecans, potentially delayed oxidation, maintaining quality in long-term refrigerated storage; mango, SL extension to 10 d, maintaining firmness and WL, reducing browning, lipid peroxidation, preserved aromatic volatiles, and antifungal activity.	[76,77,78,79]
Shellac, 10%/zein, 5%	Animal/plant	Thymol	Fresh-cut cantaloupe, efficient encapsulation with high antioxidant and antimicrobial activities.	[80]
Starch, 2–10%		-	Strawberry, extended the ripening process by up to 18 d at 20 °C.	[81]
	Plant (lima bean)	-	Sapota fruit, potential coating material compared to lima bean pod starch.	[82]
	Plant (yam bean)	Agarwood *Aetoxylon bouya* EO/calcium	Strawberry, maintained quality during storage.	[83]
Starch, 2–5%/beeswax, 33%	Plant/animal	*Eichhornia crassipes*	Fresh banana, strawberry, and fresh-cut apple, magnificent color and freshness preservation by reducing oxidation, preventing WL.	[84]
Starch, 4–5%/cellulose, 0.5%	Plant	Basil EO	Mandarin, promising for extending the SL.	[45]
Tragacanth gum, 0.6–1.5%		-	Strawberry, reduced WL and decay and preserved nutritional compounds during storage.	[13]
Wax (bees), 1–10%	Animal	Coconut oil and salicylic acid	Lemon, combined with MAP, maintained quality and shiny green color for 8 weeks; pears, prevented fruit softening.	[85,86]
Wax (bees), % n.a/zein, 0.1%	Animal/plant	Nisin	Nectarine and apple, accelerated decline of *L. monocytogenes*, did not impact the survival and growth of molds and yeasts on nectarine, but performed comparably to wax on apples.	[87]
Wax (carnauba), 1–18%		*Syzigium aromaticum* and *Mentha spicata* EO; candle soot; oleic acid; polylactic acid; and orange oil	Papaya, can act as antimicrobial; n.a. Fresh tomatoes: highest instrumental gloss and were preferred by consumers; n.a. Salacca, maintained quality and provided moderate organoleptic approval.	[88,89,90,91,92]
Whey protein, 5–15%	Animal (by-product of the cheese process)	- Mango peel extract	Peeled garlic cloves: extended the SL up to 10 d at 15 °C; Fresh-cut broccoli, improved sensory evaluation and reduced total fungi and bacterial counts.	[93,94]
Whey protein, 5%/xanthan gum, 1%	Animal/bacterial	Clove oil	Tomato, delayed senescence and maintained firmness.	[95]
Whey protein, 15%/zein, 20%	Animal/plant	-	Peeled garlic cloves, extended the SL up to 10 (whey protein) and 15 (zein) d at 15 °C.	[93]
Zein, 0.1–20%	Plant	-	Over-peeled garlic cloves, extended the SL up to 15 d at 15 °C.	[93]
	Plant (corn)	Nisin	Nectarine and apple, promising for mitigating *L.monocytogenes* contamination.	[87]

Abbreviations: AA—antioxidant activity; BI—browning index; CI—chilling injury; CMC—carboxymethylcellulose; d—days; EO—essential oil; POD—peroxidase; PPO—polyphenol oxidase; SL—shelf life; TA—titratable acidity; TPCs—total phenolic compounds; TSSs—total soluble solids; WL—weight loss; and n.a.—non-applicable/non-available.

**Table 3:** Recent bio-based edible films potentially used in fruit and vegetable food applications and their biological sources (2021–2023, ScienceDirect, Google Scholar databases).

Film Base, Base Solution Concentration	Biological Source	Incorporated Ingredient/s	Food Application: Food Product, Main Results	Reference/s
Agar, 1.1%/Arabic gum, 0.9%/konjac gum, 0.4%	Red algae/plant	Coconut oil	Cucumber, lower WL and firmness reduction at 7 °C.	[96]
Agar, 2%/maltodextrin, 2%/beeswax, 0.4–20%	Red algae/plant/animal		n.a.	[97]
Alginate, 1–3%	Macroalgae	Tannic acid; probiotic *Bacillus coagulans*/*Vitis vinifera* leaf extract (VVLE)	Activity against *E. coli*; probiotics in the film exhibited high viability during simulated digestion and showed antimicrobial and antioxidant activities; VVLE films pre-treated through ultrasonication had the highest TPC, AA, and antimicrobial properties.	[98,99,100]
Alginate, 0–3%/carrageenan, 0–3%/shellac, % n.a.	Macroalgae/animal	Cellulose nanocrystals	Cherry tomato, excellent film properties to extend the SL; shellac resulted in lower WL.	[101]
Alginate, 1.5%/cellulose, 0.5%/beeswax, 1–10%	Macroalgae/plant/animal	Anthocyanin	n.a.	[102]
Alginate, 2.5%/Arabic gum, 1%	Macroalgae/plant	Natamycin	Sweet potatoes, slowed down physiological and quality changes, with good quality after 120 days.	[103]
Alginate, 2%/konjac gum, 5%		Reuterin	Inhibition of bacteria, *B. cereus*, *C. perfringens*, and *P*. *aeruginosa*, and fungi, *F. oxysporum*, *A. alternata*, C. *gloeosporioides*, and *P. digitatum.*	[104]
Alginate, 3%/tragacanth gum, 2–14%	Macroalgae/plant	Aloe vera	n.a.	[105]
Alginate, 2%/wheat protein, 4–8%		-	n.a	[106]
Carrageenan (*ι*-), 0.5–2%/starch, 2–3.5%	Macroalgae/plant	-	n.a.	[107]
Carrageenan, 1%/whey protein, 5%	Macroalgae/animal	Probiotics (*Lactobacillus acidophilus*, *Lactobacillus plantarum*, and mixed culture)	Probiotic bacteria significantly influenced film water vapor permeability and color.	[108]
Casein, 6%/basil seed gum, 1%	Animal (bees)/plant (candelilla and carnauba)	Guar gum/gelatine-based nanogel containing lemon peel EO	Good antioxidant properties, inhibitory effect against *E. coli* and *S. aureus*, and no toxicity for endothelial cells line for 72 h.	[109]
Casein, 2–12%/pectin, 0.5–5%	Animal/plant	Egg albumin; pea protein isolate		[110,111]
Casein, 5%/starch, 5%		Pomegranate peel extract	AA, antibacterial effect against *E. coli* and *S. aureus*, slow release of bioactives in hydroalcoholic medium.	[112]
Cellulose (hydroxypropyl methyl), 1%/curdlan, 1%	Plant/bacterial	Oleic acid	n.a.	[113]
Cellulose (hydroxypropyl methyl), 4%/tamarind gum, 13.5%/zein, 8%	Plant (rice husk)	-	Cherry tomato, increased the SL, good UV barrier, transparency, and antibacterial and antioxidant activities.	[114]
Cellulose (methyl), 2%/zein, 2%		Oleic acid/thymol	Effective against *E. coli* and *S. aureus.* Incorporation of zein reduced water vapor permeability and solubility.	[115]
Cellulose, 2%/soy protein, 3%		-	n.a.	[116]
Cellulose, 1.5%/starch, 5%		Thymol	Antibacterial activity against *E. coli.*	[117]
Chitosan, 1–1.5%	Animal (crustacean shells of shrimps and fish skin)	tea tree EO	A.A. and antimicrobial properties against *L. monocytogenes* (tea tree EO 1.5%), transparency, and good UV barrier properties.	[118]
Chitosan, 1%/konjac gum, 0.3%/tragacanth gum, 0.7%	Animal/plant (acorn)	Tannic acid and ε-polylysine	n.a.	[119]
Chitosan, 1%/tamarind gum, 1–4%		Xyloglucan	Intense antimicrobial activity.	[120]
Chitosan/ulvan % n.a.		-	Ulvan extract increased physicochemical properties and AA.	[121]
Gelatine, 0.2%/Persian gum, 0.2%	Animal (scales and fins of *Cyprinus carpio:* bovine)/plant	-	n.a.	[122]
Gelatine, 3%/pectin, 3%/starch, 2%	Animal/plant	-	Antioxidant and antibacterial activities against *E. coli* and *S. aureus.*	[123]
Gellan gum, 1%/konjac gum, 0–4%	Bacterial/plant	Dihydromyricetin	Enhanced antioxidant and antimicrobial activities	[124]
Gellan gum, 0.8%/levan, 0.8%	Bacterial	-	n.a.	[125]
Gellan gum, 0–3%/quince seed gum, 0–3%/starch potato, 0–3%	Bacterial/plant		n.a.	[126]
Gluten, 3–7%/guar gum, 1–2%	Plant	-	n.a.	[127]
Persian gum, 2%	Plant	Sunflower oil	n.a.	[128]
Starch, 3–6%	Plant (corn)	-	N.a.	[129]
	Plant (pea)	Araçá extract (*Psidium myrtoides*)	Inhibits the growth of *S. aureus*	[130]
	Plant (rye)	- -	n.a.	[131]
	Plant (sago)		n.a.	[132]
Starch/polylactic acid % n.a.	Plant (cassava)/microorganism	Ferulic acid/cinnamic acid	Growth inhibition of *E. coli*, *L. innocua* and *Listeria*.	[133]
Starch, 1–5%/zein, 0.5–2%	Plant (tapioca)	Natamycin/nisin	n.a.	[134]
Wax, 0.1–0.3%/whey protein, 1.15–1.35%	Plant (candelilla)/animal	Tarbush polyphenols	n.a.	[135]
Whey protein, 5%	Animal	*Lepidium perfoliatum* gum	n.a.	[136]
Zein, 1%	Plant	Catechin and cyclodextrin	High antioxidant capacity	[137]

Abbreviations: AA—antioxidant activity; EO—essential oil; TPC—total phenolic content; WL—weight loss; and n.a.—non-applicable/non-available.

**Table 4:** Recent bio-based edible films and coatings used indistinctly in fruit and vegetable foods applications and their biological sources (2021–2023, ScienceDirect, Google Scholar databases).

Film/Coating Base, Base Solution Concentration	Biological Source	Incorporated Ingredient/s	Food Application: Food, Main Results	Reference
Alginate, 0.5%/pectin, 0.6%/xanthan gum, 0.4%	Macroalgae/plant (citrus)/bacterial	-	Fresh-cut potato, excellent preservation, coating with better effect than film heat sealing.	[138]
Agar/cellulose/gelatine/gellan/k-carrageenan/tamarind gum % n.a.	Red algae/plant/animal	-	Strawberry, reduced WL, PPO and POD activities, maintained firmness, ASA, TSS, and TA.	[16]
Casein, 2.5%/chitosan, 2%	Animal	*Origanum vulgare* L. essential oil	Cherry tomato, fungal growth inhibited for 28 days at 4 °C.	[139]
Cellulose (ligno), 2%/wheat gluten, 10%	Plant	-	Cherry, litchi, and waxberry, excellent antimicrobial properties and UV blocking.	[140]
Chitosan, 2%/levan, 2%/pullulan, 2%	Animal/bacterial (halotolerant *Bacillus* sp)/yeast	ε-polylysine	Strong inhibitory effect on two typical food-borne pathogens; WL, firmness, and TSSs of coated strawberries tended to decrease.	[141]
Corn fiber gum/chitosan (3:1)	Plant/animal	Carvacrol	Tomato and fresh-cut apple, reduced *Listeria*, *E. coli*, for up to 7 and 21 days, respectively, at 4 °C.	[142]
Corn fiber gum/whey protein (1:3)	Plant/animal			
Gellan gum, 2%	Bacterial	Cranberry extract/*Lactococcus lactis*	Fresh-cut potato and apple, probiotic film for optimal preservation; enhanced antibacterial and antioxidant activities.	[143]
Starch, 5%	Plant	Carvacrol/thymol	Mango and papaya, reduced the incidence of the fungus responsible for anthracnose.	[144]

ASA—ascorbic acid; TSSs—total soluble solids; WL—weight loss; and n.a.—non-applicable/non-available.
